# Journals, Academics, and Pandemics

**DOI:** 10.1371/journal.pmed.1000282

**Published:** 2010-05-25

**Authors:** The PLoS Medicine Editors

## Abstract

In the wake of the SARS epidemic and the H1N1 pandemic, the *PLoS Medicine* editors ask whether journal publishing is an efficient enough mechanism for information sharing.

Two articles published recently in *PLoS Medicine* highlight the problem of how to effectively share information in the wake of a rapidly spreading disease, and prompted us to ask the question “How well are journals doing?” with regard to this important goal. The answer, sadly, seems to be “not well enough.” Although the potential of the Internet for improving the dissemination of information is now taken for granted, it would seem that the attitudes of those involved in sharing this information have not kept pace with the technology. Accordingly, it is fair to ask whether the flow of information in the face of a crisis is truly enabled by publication in medical journals (even online journals) or whether we need new avenues for rapid data sharing.

An article appearing this month in *PLoS Medicine* by Weijia Xing and colleagues [1] dissected the publication of a subset of epidemiological papers during the severe acute respiratory syndrome (SARS) epidemic of 2003. Based on their findings, it would be hard to conclude that journal publication was a successful mechanism for rapidly sharing information. As the authors note, “Only 22% of the studies were submitted, 8% accepted, and 7% published during the epidemic.” What were the reasons behind these findings? The authors argue that the lack of standard methods for data collection and manuscript preparation may have played a part. In addition, despite journals' best efforts to speed up times to publication (reflected in shorter publication times compared with control articles on unrelated topics, submitted at the same time), the time to publication was over 200 days for many articles. It's not possible to know whether these delays were compounded by articles being sequentially submitted to a number of different journals before being published. But it is notable that while the 311 SARS articles were published in 137 different journals, the first ten published studies appeared in *The Lancet* (*n* = 7) and *The New England Journal of Medicine* (*n* = 3). However, the impact factors of journals publishing articles on SARS decreased significantly as time went on. Put another way, it seems that at the beginning of the epidemic, high-profile journals were willing to publish papers on SARS, but their interest waned rapidly. In addition, it is likely that, as with publishing other types of article, authors will try high-impact journals first.

Fast forward to the H1N1 pandemic of 2009–10. It's too early to carry out the same type of analysis that was done for SARS by Xing et al., but a paper we published in early April indicates that many of the same problems remain. The paper, “Association between the 2008–09 Seasonal Influenza Vaccine and Pandemic H1N1 Illness during Spring-Summer 2009: Four Observational Studies from Canada” [2] reports potentially worrying findings about the impact of seasonal flu vaccination on illness from H1N1. We're the first to admit we were not quick—we received the paper on 2 November 2009, accepted it on 1 March 2010, and published it on 6 April 2010. It's quite possible that this delay could have exacerbated the problem of making decisions about vaccination for the public health physicians and policymakers who had heard informally about some of the results and wondered if they should be changing course. At the same time, the need for careful review of these controversial data meant that we could not rush the review process. In addition, as the lead author, Danuta Skowronski, herself indicated, prior rejection from another journal added further to the delay in publication [3].

Taken together these two papers highlight an inherent limitation in the journal publication system with regard to rapid dissemination of results in a time of crisis: the processes that ensure careful evaluation come at the expense of immediate dissemination.

Journals are, of course, only one source of information for health and scientific research, and may be over-relied upon, especially in emergencies. International bodies such as WHO provided efficient regular [4] updates during both emergencies, as did local health bodies such as the Health Protection Agency in the UK [5] and the Centers for Disease Control in the US [6]. Face-to-face meetings and teleconferences provide further mechanisms for sharing of information, and when linked to sharing of resources were powerful catalysts for accelerating influenza research. PLoS itself launched an experimental site, PLoS Currents: Influenza (http://knol.google.com/k/plos-currents-influenza#) for early sharing of information after only the lightest of moderation.

Despite other mechanisms of dissemination (which need not preclude later publication in more formal peer reviewed journals) even in the face of a public health emergency, authors seem tied to publishing information first in peer-reviewed journals, possibly because they may perceive readers as reluctant to take results seriously until they have successfully emerged from review by a respected journal. It seems therefore timely to ask whether journals, and others involved in publishing, are confusing the valid role of a journal as a place of scientific record with an equally valid role, that of a news outlet? In a Canadian press report [3] on the Skowronski paper, Ross Upshur, head of the University of Toronto's Joint Centre for Bioethics, was quoted as saying “Making public health policy during a pandemic based on data most people hadn't seen was far from ideal,” adding that he believes the question of when public health priorities trump rules of scientific publication remains to be resolved: “Putting the imprimatur of a high impact peer review journal first is I think what we need to have the discussion about.”

Of course, the authors of this paper, and of all the articles published from study of the SARS epidemic and the H1N1 pandemic, would say they were not seeking such an “imprimatur” for the sake of it, but because as yet there is no widely accepted alternative to the “quality” stamp that peer review imparts. Similarly, journal editors would maintain that high-profile papers generated by such emergencies need intense scrutiny lest the public health suffer from the premature publication of unreliable results.

But in the case of a public health emergency, are these concerns enough to override the need for information—any information—however preliminary and unconfirmed? Should the whole paradigm of publishing be rethought in such instances? In the age of blogs, Twitter, and the 24-hour news cycle, are journals a realistic avenue for rapid publication at all? Instead, is there a role for data-sharing—i.e., the news outlet function—rather than traditional publication in an emergency situation? If so, who would set up data repositories and oversee them, how would authors get academic credit for their work, and how would readers and reporters learn to interpret data presented through such a system? Whatever the answers may be, it seems clear that before the next public health emergency strikes, the scientific publishing establishment needs to ask itself how it can respond in the way the world needs.
